# Primary Coenzyme Q10 Deficiency: An Update

**DOI:** 10.3390/antiox12081652

**Published:** 2023-08-21

**Authors:** David Mantle, Lauren Millichap, Jesus Castro-Marrero, Iain P. Hargreaves

**Affiliations:** 1 Pharma Nord (UK) Ltd., Morpeth NE61 2DB, UK; dmantle@pharmanord.co.uk; 2 School of Pharmacy and Biomolecular Sciences, Liverpool John Moores University, Liverpool L3 3AF, UK; l.e.millichap@ljmu.ac.uk; 3 Rheumatology Research Group, ME/CFS Research Unit, Vall d’Hebron Research Institute, Universitat Autonoma de Barcelona, 08035 Barcelona, Spain; jesus.castro@vhir.org

**Keywords:** Coenzyme Q10, primary deficiency, steroid-resistant nephrotic syndrome, encephalopathy, cardiomyopathy, ataxia

## Abstract

Coenzyme Q10 (CoQ10) has a number of vital functions in all cells, both mitochondrial and extra-mitochondrial. In addition to its key role in mitochondrial oxidative phosphorylation, CoQ10 serves as a lipid soluble antioxidant and plays an important role in fatty acid beta-oxidation and pyrimidine and lysosomal metabolism, as well as directly mediating the expression of a number of genes, including those involved in inflammation. Due to the multiplicity of roles in cell function, it is not surprising that a deficiency in CoQ10 has been implicated in the pathogenesis of a wide range of disorders. CoQ10 deficiency is broadly divided into primary and secondary types. Primary CoQ10 deficiency results from mutations in genes involved in the CoQ10 biosynthetic pathway. In man, at least 10 genes are required for the biosynthesis of functional CoQ10, a mutation in any one of which can result in a deficit in CoQ10 status. Patients may respond well to oral CoQ10 supplementation, although the condition must be recognised sufficiently early, before irreversible tissue damage has occurred. In this article, we have reviewed clinical studies (up to March 2023) relating to the identification of these deficiencies, and the therapeutic outcomes of CoQ10 supplementation; we have attempted to resolve the disparities between previous review articles regarding the usefulness or otherwise of CoQ10 supplementation in these disorders. In addition, we have highlighted several of the potential problems relating to CoQ10 supplementation in primary CoQ10 deficiency, as well as identifying unresolved issues relating to these disorders that require further research.

## 1. Introduction

Coenzyme Q10 (CoQ10) is usually described as a vitamin-like substance, although it is endogenously synthesised within most cell types. CoQ10 has a number of functions of vital importance to normal cell function; these include (i) its key role in cellular energy supply/ATP synthesis via mitochondrial oxidative phosphorylation; (ii) its role as a major endogenously synthesised lipid soluble antioxidant, protecting cellular/sub-cellular organelle membranes from free radical induced oxidative damage; (iii) its role in the metabolism of lysosomes, sulphides, amino acids, pyrimidine, and cholesterol; (iv) its role in directly mediating the expression of more than one hundred genes, including those involved in the inflammatory process [1]. Guerra and Pagliarini [2] have provided a more recent overview of the role of CoQ10 in cell metabolism. Due to the multiplicity of roles in cell function, it is not surprising that deficiency in CoQ10 has been implicated in the pathogenesis of a wide range of disorders. CoQ10 deficiency is broadly divided into primary and secondary types. Secondary CoQ10 disorders are relatively common and may occur for a variety of reasons; these include mutations in genes not directly related to the synthetic pathway, the oxidative-stress-induced reduction of CoQ10, and the effects of pharmacological agents such as statins. The role of CoQ10 deficiency and the benefits of supplementation in secondary CoQ10 disorders have been reviewed recently [3] and are not further considered in the present article.

Primary CoQ10 deficiency results from mutations in genes involved in the CoQ10 biosynthetic pathway. In man, at least 10 genes are required for the biosynthesis of functional CoQ10, a mutation in any one of which can result in a deficit in CoQ10 status [4]. Primary CoQ10 deficiency has been estimated to affect approximately 120,000 patients worldwide [5]. In general, mitochondrial disorders are not treatable; the exception is primary CoQ10 deficiency, where patients may respond well to oral CoQ10 supplementation. However, the condition must be recognised sufficiently early; once damage to critical organs such as the kidney or the nervous system is established, only minimal recovery is possible. To date, there have been two reviews relating to primary CoQ10 deficiency, by Alcazar-Fabra et al. [6] and Wang and Hekimi [7], respectively. In the review by Alcazar-Fabra et al., the overall conclusion with regard to the therapeutic outcome of CoQ10 supplementation was essentially positive, particularly with regard to steroid-resistant nephrotic syndrome. To quote the authors: “except for COQ8A patients, most individuals with primary CoQ10 deficiency show an acceptable response to CoQ10 treatment, which is usually evident after 10–20 days.” In contrast, the conclusion of the review by Wang and Hekimi was essentially negative. To quote the authors: “there is only very weak evidence for the efficacy of the treatment.” The objectives of the present article were therefore as follows: (i) to extend clinical studies (up to March 2023), on a case-by-case basis, relating to the identification of these deficiencies, and the therapeutic outcomes of CoQ10 supplementation, in an attempt to resolve the apparent discrepancy in supplementation outcomes between the above reviews; (ii) to highlight some of the potential problems relating to CoQ10 supplementation in these disorders; and (iii) to identify issues relating to primary CoQ10 deficiency that require further research, including the role of oxidative stress on clinical outcomes. 

## 2. Biosynthesis of CoQ10

**Figure 1:**
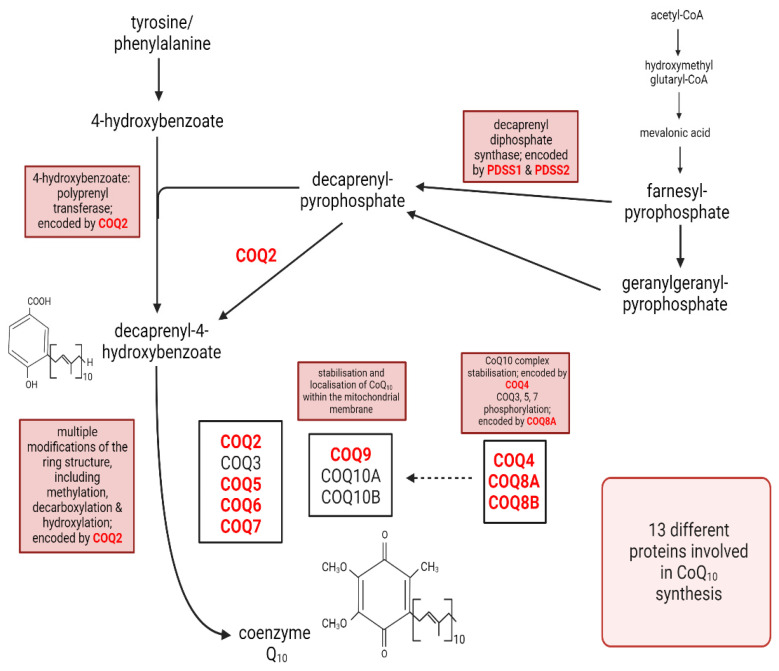
The mammalian CoQ10 biosynthetic pathway with genes harbouring known pathogenic mutations associated with primary CoQ10 deficiency shown in red.

The biosynthesis of CoQ10 is a complex multistep process (summarised in Figure 1) which takes place in various sub-cellular locations [8]. The polyisoprenoid tail is synthesised (via polyprenyl diphosphate synthase) in the cytosol via the mevalonate pathway, with attachment to the benzoquinone ring (originating from tyrosine and its metabolite 4-hydroxybenzoate) taking place within mitochondria [8]. The ring structure is then further modified via hydroxylation, methylation, and decarboxylation by a set of enzymes grouped in a complex [8].

### Genes Involved in CoQ10 Biosynthesis Pathways

At least 10 genes are required for the biosynthesis of functional CoQ10, a mutation in any one of which can result in a deficit in CoQ10 status [3]. Many of the data relating to CoQ10 biosynthesis were initially obtained from studies in yeast, with deficiencies corresponding to the above genes denoted as CoQ1 to CoQ11 (numbering refers to date order of identification) [9]. The biosynthesis of CoQ10 in yeast and man has subsequently been shown to be highly conserved [10]. With regard to the corresponding enzyme/protein gene products, COQ1 (heterotetrameric decaprenyl diphosphate synthase, comprising PDSS1 and PDSS2) is involved in the synthesis of the polyisoprenoid chain [11] and COQ2 in the condensation of the isoprenoid chain with the benzoquinone ring [12]. COQ3, COQ5, COQ6, and COQ7 are involved in concomitant methylation, decarboxylation, hydroxylation, and deamination reactions [13,14,15,16]. COQ4 is involved in the stabilisation of the CoQ10 synthetic complex [17]. COQ8A is necessary for the phosphorylation of COQs 3, 5, and 7 [18]. The COQ9 lipid-binding protein is necessary for the stabilisation of COQ7 [19], and COQ10A/COQ10B direct the localisation of CoQ10 within the mitochondrial membrane [20]. In humans, mutations in 10 of these genes have been identified to date, as described below: the corresponding gene products are, respectively, PDSS1 (phenyl diphosphate synthase subunit 1), PDSS2 (decaprenyl diphosphate synthase subunit 2), COQ2 (para-hydroxybenzoate-polyprenyl transferase), COQ4 (multienzyme complex organisation enzyme), COQ5 (methyltransferase), COQ6 (monooxygenase), COQ7 (DMG hydroxylase ADCK3 (renamed COQ8A, protein kinase), ADCK4 (renamed COQ8B, protein kinase), COQ9 (lipid-binding protein), and COQ10A/B (CoQ10 chaperone proteins).

## 3. Assessment of Primary CoQ10 Deficiency

Primary CoQ10 deficiency can be identified via both biochemical and genetic analyses. Biochemically, the determination of CoQ10 is not usually included as part of routine analysis by hospital pathology laboratories. When CoQ10 levels are measured, this is typically carried out using plasma samples, with an approximate reference range of 0.5 to 1.7 µM [21]. However, for the identification of primary CoQ10 deficiency, the tissues of choice are skeletal muscle biopsies or skin fibroblasts, since primary CoQ10 deficiency in tissues may not be manifest in plasma. Blood mononuclear cells have been suggested as an appropriate low-invasive means of assessing endogenous CoQ10 status, and these can be isolated from 5 mL of EDTA blood [22]. Furthermore, in view of the increasing association of primary CoQ10 deficiency with kidney dysfunction, the determination of urinary CoQ10 has been suggested as a reliable and non-invasive method of assessing urinary tract CoQ10 status [23]. In view of the preponderance of neurological dysfunction associated with CoQ10 deficiency, the ability to assess cerebral CoQ10 status would be of considerable diagnostic value, and cerebral spinal fluid (CSF) is considered the most appropriate surrogate to for this assessment [21]. Although tentative reference ranges have been reported, few clinical laboratories assess the CSF CoQ10 status of patients with suspected CoQ10 deficiency. The most common analytical techniques used to assess CoQ10 status are based on high-pressure liquid chromatography (HPLC) with either ultraviolet (HPLC-UV) or electrochemical (HPLC-ED) detection. The demonstration of the reduced biochemical activities of respiratory chain complexes, in particular, complexes I + III and II + III is also of relevance, since the activity of these enzyme complexes are dependent upon the endogenous CoQ10 status. Once biochemical evidence of a CoQ10 deficiency has been demonstrated, then genetic analysis can be undertaken using EDTA blood samples for gene-targeted testing and comprehensive genomic investigations [4].

## 4. Clinical Studies Relating to COQ Gene Mutations

In the following sections, clinical studies (summarised in Table 1) relating to each of the COQ biosynthetic genes COQ1–10 have been listed on a case-by-case basis in chronological order; each clinical study has been briefly summarised to include the number of patients, age of onset, symptoms, lethality, variant type, and effect of CoQ10 supplementation (where attempted). Mutations (where known) in the various COQ genes are shown in parentheses for the DNA nucleotide change and corresponding protein change, respectively.

### 4.1. COQ1

The *COQ1* gene (also known as *PDSS1/PDSS2*) encodes Subunits 1 and 2 of the enzyme hexaprenyl pyrophosphate synthetase, which catalyses the first step in CoQ10 biosynthesis, i.e., synthesis of the polyisoprenoid tail. The PDSS1 gene is located on Chromosome 10 and comprises 14 exons; PDSS2 is located on Chromosome 6 and comprises 12 exons. Mutations in PDSS1 reportedly result in steroid-resistant nephrotic syndrome, encephalopathy, and optic nerve atrophy. To date, a total of six patients with *PDSS1* mutations have been identified. These include two siblings with infantile-onset neurosensorial deafness and optic atrophy, resulting from a mutation ([c.924T>G] [p. Asp308Glu]) in the PDSS1 gene [24]; one child with a more severe birth-onset phenotype with kidney failure and death at 16 months, resulting from *PDSS1* mutations ([c.661C>T] [p. Arg221Ter] and [c661-662inST] [pArg221Leufs16]) [25]; and two sisters (aged 6 and 14 years) with sensorineural deafness and optic atrophy, resulting from a PDSS1 mutation ([c.735G>T] [p. Gln245His]) [26]. An infant with mitochondrial encephalopathy, pulmonary hypertension, and chronic distal phalangeal erythema who subsequently died aged 3 years was shown to have mutations ([c.716 T>G] [p.Val239Gly] and [c.1183C>T] [p.Arg395*]) in the PDSS1 gene [27]. Only one of the above patients was subject to CoQ10 supplementation, where CoQ10 supplementation (15 mg/kg/day) was ineffective in altering disease progression [27].

CoQ10 deficiency (COQ10D3) resulting from mutations in the *PDSS2* gene are associated particularly with neonatal/infantile-onset renal disease, with variable neurological involvement. To date, a total of seven patients with *PDSS2* mutations have been identified. The first clinical report relating to mutation of the PDSS2 gene was by Lopez et al. [28], who described an infant with nephrotic syndrome and encephalopathy, subsequently dying at 8 months. The *PDSS2* mutation in this patient involved a C→T transition at nucleotide 964, changing amino acid 322 from glutamine to a stop codon, and a C→T transition at nucleotide 1145, changing amino acid 382 from serine to leucine. Subsequent cases included a fatal PDSS2 mutation ([c485A>G] [pHis162Arg]) in a 7-month-old male infant with nephrotic syndrome, encephalomyopathy, cardiomyopathy, deafness, and retinitis pigmentosa [29], and four patients from two families with a less severe phenotype (cerebral palsy, ataxia) resulting from a *PDSS2* mutation ([c1145C>T] [pSer382Leu]) described by Sadowski et al. [30] and by Rahman et al. [31] (mutation not specified), respectively. Supplementation with CoQ10 was attempted only in the patient described by Lopez et al. [28], where the administration of CoQ10 (50 mg/day) had no effect on disease progression.

### 4.2. COQ2

The COQ2 gene is located on Chromosome 4 and comprises seven exons. *COQ2* encodes the enzyme *p*-hydroxybenzoate-polyprenyl transferase, which mediates the conjugation of the benzoquinone ring with the decaprenyl side chain in CoQ10 biosynthesis. *COQ2* mutations (causing CoQ10 deficiency COQ10D1) typically manifest as encephalopathy and nephropathy of variable severity. The first report of primary CoQ10 deficiency resulting from a *COQ2* mutation ([c890A>G] [pTyr297Cys]) was by Quinzii et al. [32], regarding two siblings with infantile nephropathy. Diomedi-Camassei et al. [33] described two patients with *COQ2* mutations, an infant with fatal encephalopathy and nephropathy who died at 6 months ([c.437G>A] [p437G>A]), and an 18-month-old infant with non-fatal nephropathy ([c590G>A] [pArg197His] and [c683A>G] [pAsn228Ser]). Supplementation with CoQ10 (30 mg/kg/day) resulted in the clinical stabilisation of the latter patient. A fatal neonatal case of nephropathy resulting from a *COQ2* mutation ([c1047delT] [pAsn351Ilefs15]) was reported by Mollet et al. [24]. Other fatal neonatal or infantile cases resulting from COQ2 mutations included a two-month-old infant with myoclonic epilepsy and hypertrophic cardiomyopathy, resulting from a [c.326G>A] [p. Ser109Asn] *COQ2* mutation, who subsequently died at 5 months despite supplementation with CoQ10 (5 mg/kg/day) [34], and a fatal case of a newborn with multi-organ failure resulting from a [c.545T>G] [p.Met182Arg] *COQ2* mutation [35]. Eroglu et al. [36] identified four infants from two families with the [c.437G→A] [p.Ser146Asn] *COQ2* mutation; in two of these patients, CoQ10 supplementation normalised renal function but did not improve neurological symptoms. Xu et al. [37] described a case of infantile nephrotic syndrome in a 14-month-old Chinese boy, resulting from *COQ2* mutations ([c.518G>A] and [c.973A>G]); the patient’s renal function was improved following CoQ10 supplementation (30 mg/kg/day). Starr et al. [38] reported three children (aged 2–10 years) with nephrotic syndrome resulting from *COQ2* mutations (two missense (p.Thr325Ala and p.Thr294Ile), and one frameshift (c.176dupT), in two of whom renal function was restored following CoQ10 supplementation (30 mg/kg/day). Wu et al. [39] reported a 6-month-old girl presenting with nephropathy with a fatal outcome, resulting from a *COQ2* mutation ([c.832 T>C] [p. Cys278Arg]). Abdelhakim et al. [40] described three siblings from a Jewish family presenting with nephropathy and retinopathy, resulting from two *COQ2* mutations ([c.288dupC] [p.(Ala97Argfs*56] and [c.376C>G] [p.(Arg126Gly]); supplementation with CoQ10 (30 mg/kg/day for 6 months) prevented further visual deterioration in these patients. Li et al. [41] identified *COQ2* mutations ([c.1058A>G] [p.Y353C] and [c.973A>G] [p.T325A]) in two Chinese infants with nephropathy; proteinuria was reduced following CoQ10 supplementation (30 mg/kg/day). Rosado-Santos et al. [42] described death in a newborn with severe foetal growth restriction, abnormal kidney function, and lissencephaly resulting from *COQ2* mutations ([c.590G>A] [p.(Arg197His] and [c.827del p.(Gly276Valfs*20]). Stallworth et al. [43] described an eight-year-old child with nephropathy requiring renal transplantation who subsequently developed progressive cone-rod dystrophy and optic atrophy, resulting from the *COQ2* variants [c683A>G] [pAsn228Ser] and [c518G>A] [parg173His]. In total, 39 *COQ2* variants in 63 patients (including previously unreported cases) have been identified to date by Drovandi et al. [44]; patients carried at least one missense variant, the most common of which was [c683A>G] [pAsn178Ser]. Supplementation with CoQ10 resulted in the partial or complete remission of proteinuria in more than half of the patients in the above study [45]. *COQ2* variants have been associated with an increased risk of developing multisystem atrophy or Parkinson’s disease [46]. 

### 4.3. COQ3

The *COQ3* gene is located on Chromosome 6 and comprises nine exons. The *COQ3* gene encodes a methyltransferase enzyme that catalyses both the O-methylation steps in CoQ10 biosynthesis. To date, there have been no clinical studies reported in the medical literature relating to *COQ3* mutations.

### 4.4. COQ4

The *COQ4* gene is located on Chromosome 9 and comprises seven exons. The *COQ4* gene encodes a multienzyme complex organisation/stabilisation enzyme localised to the matrix side of the mitochondrial inner membrane, which when deficient results in CoQ10 deficiency-7 (COQ10D7), causing childhood-onset neurodegeneration. As with other COQ mutations, the severity of disease depends on the location of the mutation. Mutations in Exons 1–4 are associated with less life-threating presentations, a late onset, responsiveness to CoQ10 therapy, and a relatively long lifespan. In contrast, pathogenic mutations in Exons 5–7 are associated with an early onset, unresponsiveness to CoQ10 therapy, and early death. The first patient with primary CoQ10 deficiency resulting from mutation of the *COQ4* gene was reported by Salviati et al. [47], a three-year-old with neurological symptoms. Oral CoQ10 supplementation (30 mg/kg/day) resulted in a significant improvement of neuromuscular symptoms.

*COQ4* mutations resulting in neurological deterioration were described in five patients ([c718C>T] [pArg240Cys]; [c433C>G] [pArg145Gly]; [c190C>T] [pPro64Ser]; [c155T>C] [pLeu52Ser]) by Brea-Calvo et al. [48], and six patients ([202G>C] [pAsp68His]; [c245T>A] [pLeu82Gln]; [c473G>A] pArg158GLN]; [c718C>T] [pArg240Cys]) by Chung et al. [49]; onset was prenatal or perinatal, with an early fatal outcome in the neonatal period or early infancy. Romero-Moya et al. [50] reported a 4-year-old with mental retardation and lethal rhabdomyolysis with a *COQ4* mutation ([c.483G>C] (E161D)). Sondheimer et al. [51] described an infant with seizures and cardiomyopathy dying in early infancy, resulting from a deletion ([c.23_33del11] [p. Val8AlafsX19]), and two missense mutations ([c.331G>T] [p. Asp111Tyr] and [c.356C>T] [p.Pro119Leu]) in the *COQ4* gene. Bosch et al. [52] identified a patient with childhood-onset spinocerebellar ataxia with stroke-like episodes, (with a different phenotype from the lethal infantile presentation described previously), resulting from a mutation ([c.230C>T] [(p. Thr77Ile]) in the COQ4 gene. Supplementation with CoQ10 (1000 mg/day) failed to prevent further stroke-like episodes.

Caglayan et al. [53] described two siblings with childhood-onset, slowly progressive ataxia resulting from a *COQ4* mutation (exon2:c.G164T:p.G55V). Supplementation with CoQ10 (200 mg/day for 1 month) resulted in significant improvement of neurological symptoms in the more severely affected sibling. Ling et al. [54] identified a *COQ4* mutation (c.370G>A) in three Asian patients presenting with infantile encephalopathy or cardiomyopathy. Lu et al. [55] found the same *COQ4* mutation (c.370 G>A (p. G124S) in a Chinese patient with Leigh syndrome (age of onset 2 months). Supplementation with CoQ10 allowed the patient to maintain a relatively stable health status. Yu et al. [56] reported on a cohort of 11 Chinese patients from nine unrelated families with the [c.370G>A] [p. Gly124Ser]) *COQ4* mutation, 5 of whom had classical neonatal-onset encephalo-cardiomyopathy, with the others having infantile onset with more heterogeneous clinical presentations. Supplementation with CoQ10 (15–40 mg/kg/day) was attempted in 7 of these 11 patients; seizure control was improved in 2 of these patients, while no benefit was noted in 5 patients who subsequently died. Chen et al. [57] described a 5-month-old patient with epileptic seizures resulting from a c.370G>A mutation in the COQ4 gene. Mero et al. [58] reported two patients each with two *COQ4* mutations ([c.577C>T] [p. Pro193Ser] and [c.718C>T] [p.Arg240Cys], and [c.284G>A] [p.Gly95Asp] and [c.305G>A] [p.Arg102], respectively), presenting with motor impairment and ataxia. 

In summary, some 28 *COQ4* variants have been identified to date, comprising a total of 44 patients; three phenotypes have been described, an early-onset phenotype with neonatal brain anomalies and epileptic encephalopathy, an intermediate phenotype with distinct stroke-like lesions, and a moderate phenotype with non-specific brain pathology and a stable disease course [59]. Patients with *COQ4* variants in Exons 1–4 (i.e., amino acid changes in the N-terminus of COQ4), and patients with the East Asian-specific c.370G>A *COQ4* variant are generally responsive to supplementation with CoQ10, whereas patients with *COQ4* variants in Exons 5–7 (amino acid changes in the C-terminus of COQ4) are generally unresponsive. 

### 4.5. COQ5

The COQ5 gene is located on Chromosome 12 and contains eight exons. The *COQ5* gene encodes the enzyme responsible for the C-methyltransferase step in CoQ10 biosynthesis (i.e., conversion of 2 methoxy-6-polyprenyl-1,4-benzoquinol to 2 methoxy-5-methyl-6-polyprenyl-1,4 benzoquinol). To date, there has been only one clinical study reported relating to mutation of the *COQ5* gene. Malicdan et al. [60] reported three female siblings from a Middle Eastern family, presenting in early childhood with seizures, cerebellar ataxia, and cognitive disability, resulting from a biallelic duplication of 9590bp in the *COQ5* gene. Supplementation with CoQ10 (15 mg/kg/day for 6 months) resulted in a significant improvement in their respective ICARS (International Cooperative Ataxia Rating Scale) scores.

### 4.6. COQ6

The *COQ6* gene is located on Chromosome 14 and comprises 14 exons. The protein encoded by this gene is a flavin-dependent monooxygenase localised to the matrix side of the inner mitochondrial membrane, which is responsible for the C5-hydroxylation of the quinone ring during CoQ10 synthesis. Mutations in this gene result in primary CoQ10 deficiency-6 (COQ10D6), an autosomal recessive disorder which typically manifests as a progressive infantile-onset steroid-resistant nephrotic syndrome resulting in end-stage renal failure, together with sensorineural deafness; individuals may also be susceptible to the development of Schwannomatosis a form of neurofibromatosis characterised by the formation of benign tumours in the nervous system. The *COQ6* mutation results in damaging effects in renal podocytes by inducing cell apoptosis, increasing cellular oxidative stress, and destroying the cytoskeleton. Whereas most forms of monogenic nephrotic syndrome in children do not respond to treatment, the identification of children with nephrotic syndrome resulting from defective CoQ10 biosynthesis is vital, since such individuals may respond to CoQ10 supplementation. Primary CoQ10 deficiency due to *COQ6* mutations should be considered in children presenting with both steroid-resistant nephrotic syndrome and sensorineural hearing loss. 

Heeringa et al. [61] first identified six different *COQ6* mutations ([c484C>T] [pArg162]; [c564G>A] [pTrp188]; [c763G>A] [pGly255Arg]; [c1058C>A pAla353Asp]; [ c1341G>A] [pTrp447]; [c12383delG] [pGln461fs478]) in 13 individuals from seven families by homozygosity mapping. Each mutation was linked to early-onset (1–2 years) steroid-resistant nephrotic syndrome and sensorineural deafness, with some patients responding favourably (reduced proteinuria, improved hearing) to oral CoQ10 supplementation (50 mg twice daily). However, such patients may not benefit from CoQ10 therapy when severe renal and neurological damage is established; thus, an early and accurate diagnosis of COQ10D6 and simultaneous CoQ10 intervention are critical in improving prognosis.

Sadowski et al. [30] described six patients with infancy/childhood onset of nephropathy resulting from COQ6 mutations ([c1058C>A] [pAla353Asp]; [c1154A>C] [pAsp385Ala]; [c1235A>G] [pTyr412Cys]). Park et al. [62] identified COQ6 mutations ([c189 191 del GAA] [pLys64del]; [c686A>C] [pGln229Pro]; [c782C>T] [pPro26Leu]) in a series of six children (age of onset 15–47 months), all of whom progressed to end-stage renal disease requiring transplantation. Li et al. [63] diagnosed a child at 1 year with a mutation ([c.1078C>T] [p.Arg360Trp]]) in the *COQ6* gene; proteinuria was subsequently completely resolved following supplementation with CoQ10 (30 mg/kg per day for 3 months). Cao et al. [64] described a case of infantile nephrotic syndrome resulting from a *COQ6* mutation ([c1078C>T] [pArg360Trp]); supplementation with CoQ10 (30 mg/kg/day) over a period of 3 months restored normal renal function. Song et al. [65] reported a 16-year-old presenting with proteinuria and subsequently identified as having a COQ6 mutation ([c.G41A] [p.W14X]). Supplementation with CoQ10 over a 6-month period reduced the 24 h urinary protein by approximately 50%. 

Stanczyk et al. [66] described a 4-year-old presenting with steroid-resistant glomerulopathy with *COQ6* mutations ([c.1078C>T] [p.Arg360Trp]]; [c.804delC] [pLeu269Trpfs13]). Supplementation with CoQ10 (30 mg/kg per day for one month resulted in complete symptomatic remission. Yildirim et al. [67] reported on a 7-year-old girl diagnosed with steroid-resistant nephrotic syndrome and sensorineural deafness, requiring haemodialysis and subsequent renal transplantation (mutation [c.1058C>A] [rs397514479] in Exon 9 of *COQ6*). 

Perrin et al. [68] described a *COQ6* mutation in a patient with renal disease (requiring transplantation) and deafness, who also developed loss of vision. In this case, supplementation with the CoQ10 analogue idebenone, which has been used for the treatment of other types of optic neuropathy, improved visual impairment without affecting deafness. Wang et al. [69] identified two Chinese siblings with COQ10D6 who presented with severe metabolic acidosis, proteinuria, hypoalbuminemia, growth retardation, and muscle hypotonia and died in early infancy (mutations: [c.249C>G] [p.Tyr83Ter] in Exon 2 and [c.1381C>T] [p.Gln461Ter] in Exon 12 of *COQ6*). A 19-month-old patient with cardiomyopathy was found to have the COQ6 variant [c763G>A] [p.Gly255Arg]; the patient died from cardiorespiratory failure before CoQ10 supplementation could be started [70]. Nam et al. [71] identified 12 children from 11 unrelated Korean families resulting from *COQ6* mutations (c.189_191delGAA (p.Lys64del), c.484C>T (p.Arg162*), c.686A>C (p.Gln229Pro), and c.782C>T (p.Pro261Leu)). The effects of CoQ10 supplementation (30 mg/kg) on hearing loss were further investigated in seven of these children (mean age 7.2 years at start of CoQ10 treatment). In terms of the prevention of hearing loss, approximately 50% of patients responded well to treatment with CoQ10; those with the c.686A>C variant responded poorly to CoQ10 supplementation, but those with c.189_191delGAA or c.782C>T variants responded well. 

The total number of patients identified with a *COQ6*-related primary CoQ10 deficiency is currently 45, with 14 of these responding to CoQ10 supplementation (where attempted).

### 4.7. COQ7

The *COQ7* gene is located on Chromosome 16 and comprises 11 exons. The *COQ7* gene encodes the enzyme 5-demethoxyubiquinone hydroxylase, which catalyses the hydroxylation of converting demethoxyubiquinone to 5-hydroxy-ubiquinone. Mutations in the *COQ7* gene result in primary CoQ10 deficiency-8 (COQ10D8). To date, four clinical cases relating to mutations in the *COQ7* gene have been reported. The first case was described by Freyer et al. [72], a 9-year-old Syrian boy with progressive encephalo-neuro-nephro-cardiopathy resulting from a missense mutation ([c.422T>A] [p.Val141Glu]) in Exon 4 of the COQ7 gene. CoQ10 levels were severely decreased in the skeletal muscle and fibroblasts of the patient. The authors demonstrated that the coenzyme Q analogue 2,4-dihydroxybenzoic acid was able to specifically bypass the COQ7 deficiency, increase cellular coenzyme Q levels, and rescue the biochemical defect in patient fibroblasts. The second case was reported by Wang et al. [73], a 6-year-old girl presenting with spasticity and bilateral sensorineural hearing loss resulting from two mutations ([c.308C>T] [p. Thr103Met] and [c.332T>C] [p.Leu111Pro]) in the *COQ7* gene. Only a moderate decrease in CoQ10 levels were found in skin fibroblast cells from this patient. The third case was reported by Kwong et al. [74], a Chinese boy presenting with encephalo-myo-nephro-cardiopathy, with a fatal outcome at one year of age, resulting from a frameshift [c.599_600delinsTAATGCATC] [p.(Lys200Ilefs*56] and a missense substitution [c.319C>T] [p.(Arg107Trp]) in the *COQ7* gene. This patient showed a poor response to supplementation with CoQ10 (20 mg/kg/day). For the fourth case, Wang et al. [75] identified a 4-year-old Turkish boy with a mutation ([c.161G>A] [p. Arg54Gln]) in Exon 2 of the *COQ7* gene, presenting with hypotonia, difficulty walking, motor developmental delay, ataxia, and spasticity. The patient’s skin fibroblasts showed a 45% reduction in the CoQ10 level with a concomitant reduction in mitochondrial respiratory capacity, which could be rescued using a CoQ10/caspofungin formulation.

### 4.8. COQ8A

The *COQ8A* gene is located on Chromosome 1 and comprises 18 exons. The *COQ8A* gene, also known as *ADCK3* or *CABC1*, encodes an atypical protein kinase. Patients with mutations in this gene typically develop childhood-onset cerebellar atrophy and ataxia, and have a decreased CoQ10 content in their muscle, fibroblast, and lymphoblast cells. Mollet et al. [76] described four children (aged 18 months to 3 years) from three unrelated families with seizures and cerebellar atrophy resulting from various *COQ8A* mutations ([c1655G>A] [Glu551Lys]; [p 815G>T] [Gly272Val]; [c636C>T] [Arg213Trp]; [c815G>A] [Gly272Asp]). Supplementation with CoQ10 (10 mg/kg/day) did not result in clinical improvement in these patients. Lagier-Tourenne et al. [77] identified six patients with childhood-onset cerebellar atrophy and ataxia resulting from *COQ8A* mutations ([c500521del22insTTG] [pGln167Leufs36]; [c993C>T] [pLys314Gln360del]; c17501752delACC] [pThr584del]; [c16456G>A] [pGly549Ser]; [c1541A>G] [pTyr514Cys]; [c139 + 2T>C] [pAsp420Trpfs40]); no CoQ10 supplementation was attempted in any of these patients. Blumkin et al. [78] reported two sisters with *COQ8A* mutations ([c805C>G] [pPro602Arg] and [c1750 1752 delACC] [pThr584del]) with a variable phenotype; one sister had childhood-onset severe progressive ataxia which was partially resolved following CoQ10 supplementation (20 mg/kg/day), whilst the second sister had only mild dysarthria. Hikmat et al. [79] reported three Norwegian patients with childhood-onset cerebellar ataxia and epilepsy, with stroke-like episodes, resulting from *COQ8A* mutations ([c895C>T] [pArg229Trp]; [c1732T>G] [pPhe578Val]). Two children with cerebellar atrophy and ataxia resulting from *COQ8A* mutations ([c803T>C] [pLeu227Pro] and [c1506 + 1G>A] [pVal503Metfs21]) were described by Jacobsen et al. [80]; supplementation with CoQ10 (20 mg/kg/day) over a 12-month period resulted in an improvement in ataxia and mental capacity in both siblings. Chang et al. [18] carried out a literature review of 22 cases with *COQ8A* mutations for which CoQ10 supplementation had been attempted; 11 patients showed clinical benefit, with ataxia being the symptom showing greatest response. On this basis, the authors recommended a CoQ10 dosage regime of at least 15 mg/kg/day for 6 months for patients with *COQ8A*-related ataxia. Nair et al. [81] detailed a 5-year-old girl with severe intellectual disability and ataxia resulting from a *COQ8A* mutation ([c1534C>T] [pArg512Trp]). Schirinzi et al. [82] described an improvement in motor performance following CoQ10 supplementation (15 mg/kg/day) over a period of 6–12 months in four children with ataxia. Uccella et al. [83] reported a child with early-onset ataxia resulting from a COQ8 mutation (c.901C>T;c.589-3C>G), where CoQ10 supplementation slowed down disease progression. Paprocka et al. [84] described a 22-month-old girl with cerebellar ataxia and developmental regression resulting from a *COQ8A* mutation ([c.811C>T] [p.Arg271Cys] who showed improved communication and growth following supplementation with CoQ10 (300 mg/day for 3 months). Degerliyurt et al. [85] reported on a 16-year-old patient with ataxia, cerebellar atrophy, and cardiomyopathy resulting from a *COQ8A* mutation ([c901 C>T] [p. Arg301Trp]); the authors noted that supplementation with CoQ10 was started at too late a stage in disease to prevent the death of the patient. The most comprehensive study on patients with *COQ8A* mutations was carried out by Traschutz et al. [86], who assessed 64 patients (including 39 previously unreported) from 51 families across 16 different countries. This work incorporates results from previous studies by Horvath et al. [87], Mignot et al. [88], and Mallaret et al. [89]. Cerebellar ataxia was evident in all patients, with a mean age of onset of 7 years. A total of 44 *COQ8A* variants were identified in these patients, comprising 26 missenses and 18 loss-of-function variants (10 frameshift, 4 stop, 3 canonical splice, and 1 in-frame deletion), distributed across the entire *COQ8A* gene. Out of a total of 30 patients supplemented with CoQ10 (mean daily dose of 11 mg/kg/day), approximately half of these patients were classed as responders to treatment.

### 4.9. COQ8B

The *COQ8B* gene is located on Chromosome 19 and comprises 16 exons. The *COQ8B* gene (also known as *ADCK4*) similarly encodes an atypical protein kinase, variants in which result in steroid-resistant nephrotic syndrome with variable neurological involvement (presenting mainly in childhood or adolescence). To date, 99 patients with *COQ8B* mutations have been identified. Ashraf et al. [90] reported a patient with nephropathy resulting from a homozygous *COQ8B* frameshift mutation, who subsequently had partial remission following CoQ10 supplementation. Korkmaz et al. [91] identified 26 adolescent patients with nephropathy resulting from *COQ8B* mutations ([c293T>G] [pLeu98Arg]; [c332C>T] [[pArg178Trp]; [c929C>T [pPro301Leu]; [c748G>A] [pAsp250Asn]; [c1199dupA] [pHis400Glnfs11]; [c1339dupG] [pGlu447Glyfs10]). Two patients in the early stage of renal damage showed improved proteinuria following CoQ10 supplementation (15 mg/kg/day) over a 6-week period. Atmaca et al. [92] reported significant improvement in proteinuria in eight patients with *COQ8B*-related nephropathy, following CoQ10 supplementation. Feng et al. [93] identified two children with proteinuria resulting from a *COQ8B* mutation, one of whom showed a good response to CoQ10 therapy (15–30 mg/kg/day). Song et al. [94] identified 20 Chinese children from 17 families with renal disease resulting from *COQ8B* mutations, with the c.737G>A (p. S246N) and c.748G>A (p.D250H) variants being most prevalent. In a trial group of five patients, supplementation with CoQ10 (15–30 mg/kg/day) reduced proteinuria. 

### 4.10. COQ9

The *COQ9* gene is located on Chromosome 16 and comprises nine exons. The *COQ9* gene encodes a lipid-binding protein that is responsible, via its interaction with other COQ-encoded enzymes (particularly COQ7), for stabilising the COQ10 biosynthetic complex. Mutations in this gene (responsible for COQ10D5) have been reported in seven patients to date. Duncan et al. [95] reported an infant with multisystem disease, including intractable seizures, global developmental delay, hypertrophic cardiomyopathy, and renal tubular dysfunction, who died at 2 years of age despite supplementation with CoQ10 (up to 300 mg/day). This patient was found to have the *COQ9* mutation [c.730C>T] [p.Arg244]. Danhauser et al. [96] identified an infant of Turkish origin with fatal neonatal lactic acidosis and encephalopathy, resulting from a *COQ9* mutation ([c.521 + 1del] [p.Ser127-Arg202del]). Smith et al. [97] described four siblings with multisystem disease, two of whom died soon after birth with the *COQ9* variants [c711 + 3G.A] [pAla203Asp237del] and [c521 + 2T>C] [pSer127Arg202del]. Olgac et al. [98] described a 9-month-old girl of Pakistani origin presenting with microcephaly and seizures, resulting from a COQ9 mutation ([c.384delG] [Gly129Valfs*17]). There was no improvement in neurological symptoms following supplementation with CoQ10 (5–50 mg/kg/day).

### 4.11. COQ10A and COQ10B

The *COQ10A* gene is located on Chromosome 12 and comprises six exons; *COQ10B* is located on Chromosome 2 and comprises six exons. The *COQ10A* and *COQ10B* genes encode lipid-binding proteins which act as molecular chaperones, directing CoQ10 molecules to their final placement within membranes. COQ10A and COQ10B proteins are expressed in all tissues, although COQ10A is predominantly expressed in the heart and skeletal muscle. Polymorphisms in *COQ10A* or *COQ10B* have been implicated in predisposing patients to statin-associated myopathy [95]. To date, there have been no clinical studies published relating to *COQ10A* or *COQ10B* variants.

### 4.12. HPDL Deficiency

Whilst not part of the *COQ1–COQ10* gene mutation series described above, the enzyme 4-hydroxyphenylpyruvate dioxygenase-like protein (HPDL) has also been shown to have a role in CoQ10 biosynthesis [99]. Patients with HPDL deficiency have been reported by Husain et al. (17 individuals, [100]), Wiessner et al. (34 individuals, [101]), Wang et al. (1 individual, [102]), and Micule et al. (2 individuals, [103]). The patient ages in these studies ranged from 6 months to 39 years; the clinical presentation typically included development delay, seizures, and spasticity. Treatment of patients with HPDL mutations with 4-hydroxymandelate (a metabolite of HPDL) or CoQ10 may stabilise or ameliorate some of these symptoms.

**Table 1:** Summary of publications relating to gene and associated clinical features in primary CoQ10 deficiency.

Author	Gene	Condition	Number of Patients	CoQ10 Dose and Comment
Mollet et al. [24]	***COQ1*** ***(PDSS1)***	Infantile neurosensorial deafness and optic atrophy	2	
Vasta et al. [25]		Kidney failure and death at 16 months	1	
Nardecchia et al. [26]		Sensorineural deafness and optic atrophy (ages 6 and 14 years)	2	
Bellusci et al. [27]		Numerous morbidities; death aged 3 years	1	15 mg/kg/day No effect on disease progression
Lopez et al. [28]	***COQ1*** ***(PDSS2*** **)**	Nephrotic syndrome and encephalopathy; died at 8 months	1	50 mg/day. No effect on disease progression
Ivanyi et al. [29]		Numerous morbidities; death at 7 months	1	
Sadowski et al. [30]		Less severe morbidities	2	
Rahman et al. [31]		Infantile-onset encephalopathy	2	
Quinzii et al. [32]	***COQ2***	Infantile nephropathy	2	
Diomedi-Camassei et al. [33]		Encephalopathy and nephropathy in the first patient who died at 6 months. Non-fatal nephropathy in a second patient.	2	30 mg/kg/day clinical stabilisation in second patient
Mollet et al. [24]		Fatal neonatal nephropathy	1	
Scalais et al. [34]		Myoclonic epilepsy and hypertrophic cardiomyopathy; died at 5 months	1	5 mg/kg/day did not prevent fatality
Desbats et al. [35]		Newborn multiorgan failure and death	1	
Eroglu et al. [36]		Infantile renal and neurological symptoms	4	Supplement normalised renal function in 2 patients
Xu et al. [37]		Infantile nephrotic syndrome	1	30 mg/kg/day improved renal function
Starr et al. [38]		Nephrotic syndrome (patients 2–10 years old)	3	30 mg/kg/day restored renal function in 2 patients
Wu et al. [39]		Fatal neuropathy at 6 months	1	
Abdelhakim et al. [40]		Nephropathy and retinopathy	3	Visual deterioration prevented after 30 mg/kg/day CoQ10 for 6 months
Li et al. [41]		Infantile nephropathy	2	Proteinuria reduced after 30 mg/Kg/day CoQ10
Rosado-Santos et al. [42]		Newborn fatality with severe fetal growth restriction	1	
Stallworth et al. [43]		8-year-old with nephropathy and optic atrophy	1	
Drovandi et al. [44,45]		Review articles to include many of the above studies relating to COQ2, plus some previously unreported cases.	63	Partial or complete remission of proteinuria in more than half of patients after CoQ10
No reports identified	***COQ3***			
Salviati et al. [47]	***COQ4***	3-year-old with neuromuscular symptoms	1	Significant symptomatic improvement after 30 mg/kg/day CoQ10
Brea-Calvo et al. [48]		Neurological deterioration with neonatal or early infancy fatality	5	
Chung et al. [49]		Neurological deterioration and early deaths	6	
Romero-Moya et al. [50]		Mental retardation and lethal rhabdomyolysis aged 4	1	
Sondheimer et al. [51]		Seizures, cardiomyopathy, and death in infancy	1	
Bosch et al. [52]		Infantile spinocerebellar ataxia and stroke-like episodes	1	No improvement after 1000 mg/day CoQ10
Caglayan et al. [53]		Childhood-onset slow progressive ataxia	2	One sibling improved after 200 mg/day CoQ10 for 1 month
Ling et al. [54]		Infantile encephalopathy or cardiomyopathy	3	
Lu et al. [55]		2-month-old with Leigh syndrome	1	Patient stabilised after CoQ10
Yu et al. [56]		Numerous morbidities with neonatal or infantile onset	11	Seizure control improved in 2 patients after 15–40 mg/kg/day CoQ10; no benefit in 5 patients who subsequently died
Chen et al. [57]		5-month-old with epileptic seizures	1	
Mero et al. [58]		Motor impairment and ataxia	2	
Malicdan et al. [60]	***COQ5***	Seizures, ataxia and cognitive disability in early childhood	3	Symptomatic improvement in all cases after 15 mg/kg/day CoQ10 for 6 months
Heeringa et al. [61]	***COQ6***	Early-onset (1–2 years) nephrotic and sensory syndromes	13	Symptomatic improvement in some patients with 100 mg/day CoQ10
Sadowski et al. [30]		Infancy/early childhood-onset nephropathy	6	
Park et al. [62]		Renal disease (onset 15–47 months) requiring transplant	6	
Li et al. [63]		1-year-old with proteinuria	1	Proteinuria completely resolved after 30 mg/day CoQ10 for 3 months
Cao et al. [64]		Infantile nephrotic syndrome	1	Normal renal function restored after 30 mg/kg/day CoQ10 for 3 months
Song et al. [65]		Proteinuria in a 16-year-old	1	50% reduction in proteinuria after CoQ10
Stanczyk et al. [66]		Glomerulopathy in a 4-year-old	1	Complete symptomatic remission after 30 mg/kg/day CoQ10 for 1 month
Yildirim et al. [67]		Nephrotic syndrome and sensorineural deafness in a 7-year-old	1	
Perrin et al. [68]		Renal disease, deafness, and optic neuropathy	1	The CoQ10 analogue, idebenone, improved vision
Wang et al. [69]		Numerous morbidities: died in infancy	2	
Leeuwen et al. [70]		19-month-old with fatal cardiomyopathy	1	
Nam et al. [71]		Patients (<18 years) with nephropathy and deafness	12	Hearing loss in some patients responded well after 30 mg/kg CoQ10
Freyer et al. [72]	***COQ7***	Numerous morbidities in a 9-year-old; muscle CoQ10 levels severely decreased	1	
Wang et al. [73]		Spasticity and sensorineural hearing loss in a 6-year-old; moderate decrease in CoQ10 levels	1	
Kwong et al. [74]		Fatal morbidities at 1 year	1	Poor response to treatment after 10 mg/kg/day CoQ10
Wang et al. [75]		4-year-old with numerous morbidities: 45% reduction in fibroblast CoQ10 levels	1	
Mollet et al. [76]	***COQ8A***	Seizures and cerebellar atrophy (ages 18–36 months)	4	Not improved after 15 mg/kg/day CoQ10
Lagier-Tourenne et al. [77]		Childhood-onset cerebellar atrophy and ataxia	6	
Blumkin et al. [78]		Ataxia and mild dysarthria	2	Ataxia partially resolved after 20 mg/kg/day CoQ10
Hikmat et al. [79]		Childhood-onset cerebellar ataxia and epilepsy	3	
Jacobsen et al. [80]		Childhood-onset cerebellar atrophy and ataxia	2	Improvement in ataxia and mental capacity after 20 mg/kg/day CoQ10
Nair et al. [81]		5-year-old with intellectual disability and ataxia	1	Improved motor performance following 15 mg/kg/day CoQ10
Schirinzi et al. [82]		Ataxia in patients aged 4–12 months	4	
Uccella et al. [83]		Childhood-onset ataxia	1	Disease progression slowed after CoQ10
Paprocka et al. [84]		Cerebellar ataxia and developmental regression in a 22-month-old	1	Improved communication and growth after 300 mg/day CoQ10
Degerliyurt et al. [85]		16-year-old with ataxia, cerebellar atrophy, and cardiomyopathy	1	Treatment with CoQ10 started at too late a stage to prevent death of the patient.
Traschutz et al. [86]		Review incorporating some previously published data; cerebellar ataxia in all patients with a mean onset age of 7 years	64	50% of patients responded after a mean dose of 11 mg/Kg/day CoQ10
Ashraf et al. [90]	***COQ8B***	Nephropathy	1	Partial remission after CoQ10
Korkmaz et al. [91]		Adolescent nephropathy	26	Improved proteinuria after 15 mg/kg/day CoQ10 in 2 patients
Atmaca et al. [92]		Nephropathy	8	Improved proteinuria after CoQ10
Feng et al. [93]		Patients aged 9 months and 11 years with proteinuria	2	Younger subject showed a good response after 15 mg/kg/day CoQ10
Song et al. [94]		Patients aged 1–18 years with renal disease	20	Reduced proteinuria in a trial group of 5 subjects after 15–30 mg/kg/day CoQ10
Duncan et al. [95]	***COQ9***	Multiple morbidities; died aged 2 years	1	Up to 300 mg/day CoQ10 did not prevent fatality
Danhauser et al. [96]		Fatal neonatal lactic acidosis and encephalopathy	1	
Smith et al. [97]		Multisysystem disease in 4 siblings, 2 of whom died soon after birth	4	
Olgac et al. [98]		Microcephaly and seizures in a 9-month-old	1	No improvement after 5–50 mg/kg/day CoQ10
No reports identified	***COQ*** ***10A*** **and *10B***			

## 5. Discussion

Primary CoQ10 deficiency resulting from COQ gene mutations is associated with a heterogeneous spectrum of clinical phenotypes; however, from the data presented above (summarized in Table 1), the following generalisations may be made: (i) the disorder typically has a neonatal, infantile, or childhood age of onset; (ii) patients typically present with neurological dysfunction (encephalopathy, psychomotor delay, cerebellar atrophy/ataxia, optic atrophy), nephropathy, cardiomyopathy, or any combination thereof; (iii) the outcome for patients is typically either serious disability or fatality. To date, some 300 patients with primary CoQ10 deficiency have been identified, in approximately 100 of whom CoQ10 supplementation was attempted, as described in the present review. Some of the above syndromes can be rescued by oral supplementation with CoQ10, particularly when diagnosed sufficiently early; however, the response to therapy depends on which COQ gene has been affected, and the particular location of the mutation within each of the respective genes. Steroid-resistant nephrotic syndrome resulting from COQ mutations appears to be particularly amenable to treatment via CoQ10 supplementation. For example, Drovandi et al. [44] assessed the longer term efficacy of supplemental CoQ10 in a series of 40 patients (less than 18 years) with chronic kidney disease (Stage 1–4) compared to an untreated cohort matched by genotype, age, kidney function, and proteinuria. Supplementation with CoQ10 resulted in a substantial and significant sustained reduction in proteinuria (by 88%) after 12 months. Complete remission of proteinuria was more frequently observed in patients with renal disease resulting from COQ6 mutations. Supplementation with CoQ10 resulted in a significantly better preservation of kidney function (5-year kidney failure-free survival 62% vs. 19%), together with improvements in neurological manifestations and general health. The authors of this study consider that patients with this type of primary CoQ10 deficiency should receive early and life-long supplementation with CoQ10 to decrease kidney disease progression and prevent further damage to other organs. As noted in the Introduction to this article, there have been two previous reviews relating to primary CoQ10 deficiency, by Alcazar-Fabra et al. [6] and Wang and Hekimi [7], that differed in their conclusions regarding the efficacy of CoQ10 supplementation in primary CoQ10-deficiency disorders. On the basis of the data presented in this article, we would concur with the conclusion of Alcazar-Fabra et al. that CoQ10 supplementation should be attempted at the earliest possible stage in patients with primary CoQ10 deficiencies. In patients who have died, the lack of response to CoQ10 supplementation results when irreversible tissue damage has already occurred prior to diagnosis; in such patients, CoQ10 deficiency in cultured fibroblasts can typically be rescued following CoQ10 supplementation.

### 5.1. Potential Issues Relating to CoQ10 Supplementation

In addition to the type of genetic mutations described above, the response to therapy can depend on a number of factors relating to the supplement itself. In contrast to prescription-type drugs which require marketing authorisation by regulatory authorities, products classed as food supplements are not subject to the same regulatory standards. Thus, there is no regulatory requirement for the manufacturers of CoQ10 supplements to guarantee the quality, efficacy, and safety of their products. Of the 38 clinical studies identified in this article in which supplemental CoQ10 had been used to treat patients with primary CoQ10 deficiency, only 2 studies specified the manufacturer of the supplement used. On this basis, it is difficult to judge the outcome of such studies in which poor quality supplements may have been used.

In addition to the above issue, the bioavailability of the supplement formulation, dose, and duration of treatment may affect the outcome of clinical studies. As a result of the particular chemical structure of CoQ10 (one of the most hydrophobic naturally occurring substances), the bioavailability of supplemental CoQ10 is low. CoQ10 is produced via a yeast fermentation process in the form of polymorphic crystals, which cannot be absorbed from the digestive tract. CoQ10 can be absorbed only as individual molecules, as noted above. To be effective as a supplement, the CoQ10 crystals must therefore be dissociated first into individual CoQ10 molecules prior to absorption. The above process involves changing the shape of the CoQ10 crystals in such a way as to increase the ratio of the crystals’ surface area to the volume, thus making it easier for the crystals to dissolve into single molecules at body temperature. CoQ10 formulations which have not been subjected to CoQ10 crystal dispersion may have their bioavailability reduced by 75% [104]. There is currently no consensus as to the dosage of CoQ10 for the treatment of primary CoQ10 deficiency disorders, although a dose of 15–30 mg/kg/day has been used in a number of studies. The relationship between the level of CoQ10 in the bloodstream to that in the various tissues has been questioned. The mechanism by which CoQ10 is transported from the blood into tissues is not understood, but it has been postulated that a high level of CoQ10 in the blood is required to drive CoQ10 into the cells of tissues; this in turn suggests a lack of specific transporters of CoQ10, with access to cells reliant on simple diffusion, which is slow and depends on the concentration gradient provided by a high circulating dose [105]. Given these potential problems with supplement quality and bioavailability, it is perhaps surprising that the clinical studies described in the present article have been as successful; the use of a CoQ10 supplement manufactured to marketing authorisation standards would be expected to increase the success of such clinical studies considerably.

### 5.2. Future Directions on Unresolved Issues

As noted above, the bioavailability of orally supplemented CoQ10 is inherently low. The possibility of administering CoQ10 via alternative routes, for example intravenous, intraperitoneal, intramuscular, or subcutaneous injection, has been reviewed by Mantle et al. [106]. These methods, together with the possibility of developing a CoQ10 inhaler, remain as areas for future research. 

In neonatal fatalities, there is a rationale for CoQ10 supplementation of at-risk mothers during pregnancy, in order to reduce tissue damage during foetal development. However, it is well known that the placenta acts as a selective barrier between mother and foetus; whether supplemental CoQ10 can cross the placental barrier in humans has yet to be established, and it remains an issue for further research.

It is also of note that the correction of CoQ10 deficiency in cultured fibroblasts or yeast models, but not in patients with neurological symptoms, may indicate an insufficient uptake of exogenous CoQ10 across the blood–brain barrier in these patients. Establishing whether supplemental CoQ10 can cross the blood–brain barrier in man is another issue requiring further research; as noted in the recent review by Munch et al. [107], the use of 31P-MRSI (magnetic resonance spectroscopic imaging) or PET (positron emission tomography) imaging of labelled CoQ10 may help to further elucidate this issue.

In addition, there is a need to establish a method of determining CoQ10 levels during newborn screening to enable the identification and treatment of primary CoQ10-deficient patients prior to the maturation of the blood–brain barrier. There is presently no consensus as to how this should be performed, although in principle CoQ10 levels can be determined in dried blood spots taken during screening for phenylketonuria [108]; such general screening would enable the identification of cases resulting from new gene mutations, as well as individuals from families with a history of primary CoQ10 deficiency 

Finally, there is the question of whether the administration of supplements in addition to CoQ10 might be of benefit in these disorders. The rationale underlying this approach has been described previously with regard to the treatment of neurological disorders; the supplements considered include those of relevance to cellular energy generation and antioxidant activity such as B-vitamins/NADH, L-carnitine, vitamin D, and alpha-lipoic acid [109]. 

### 5.3. Mitochondrial Dysfunction and Oxidative Stress in Primary CoQ10 Deficiency

With regard to the relative importance of mitochondrial respiratory chain defects and oxidative stress in the pathogenesis of CoQ10, there is presently no clear relationship between the type of CoQ mutation, the level of CoQ10 deficiency or the degree of oxidative stress in patients with primary CoQ10 deficiencies. Work in cultured skin fibroblasts with primary CoQ10 deficiencies has shown that severe CoQ10 deficiency affects ATP synthesis to a greater degree than ROS generation, whereas moderate CoQ10 deficiency leads to a greater degree of ROS generation with relatively unaffected energy synthesis [110]. This study suggested a variable effect of CoQ10 deficiency on mitochondrial oxidative stress; whereby severe (<20% residual CoQ10 status) and moderate (>60% residual CoQ10) deficiencies caused low levels of oxidative stress as measured by mitochondrial ROS generation and cellular lipid peroxidation markers, malondialdehyde and 4-hydroxyalkenals. However, an intermediate deficiency (30–40% of residual CoQ10 status) resulted in an increase in the level of mitochondrial oxidative stress. 

It is uncertain thus far as to why an intermediate level of CoQ10 deficiency has the capacity to induce more oxidative stress than a more profound CoQ10 deficiency. However, an intermediate CoQ10 deficiency may result an inefficient transfer of electrons from ETC complex I and II to III, resulting in an increased electron leakage to molecular oxygen and superoxide generation, as well as the enhanced production of the unstable semiquinone isoform of CoQ10, a major site of ROS production [111].

A more profound state of CoQ10 deficiency would decrease electron transfer in the ETC and, consequently, the possibility of electron leakage from the chain. Interestingly, in a study by Quinzii et al. [110], fibroblasts with COQ9 and PDSS2 mutations were found to have the lowest level of residual CoQ10 status (18% and 22% of control levels, respectively), whereas fibroblasts harbouring a COQ2 mutation had an intermediate level of CoQ10 deficiency (36–43% of control levels) and showed the highest level of oxidative stress generation. In an “in vitro” human neuronal cell model of CoQ10 deficiency, a 24% decrease in the level of cellular CoQ10 (76% residual CoQ10 status), which was also accompanied by a global loss of ETC enzyme activities, was found to cause a four-fold increase in the level of mitochondrial ROS compared to control levels [112]. This result indicates the potential vulnerability of neurons to a small deficit in CoQ10 status as compared to fibroblasts; in the latter cells, evidence of ROS generation and oxidative stress generation was not apparent until the level of cellular CoQ10 decreased by 60–70% of control levels. The reason for the disparity between the two cell types is as yet unknown but may again reflect variations in cellular antioxidant status. Importantly, these results indicate that the degree of CoQ10 deficiency required to induce oxidative stress may vary between cell types. Neurons appear especially vulnerable to a small deficit in the level of CoQ10, and this may support the importance of assessing and monitoring the cerebral CoQ10 status in patients with suspected primary CoQ10 deficiencies. 

### 5.4. Therapeutic Cosupplementation in Primary CoQ10 Deficiency

The question of whether the co-administration of antioxidants could be of benefit in primary CoQ10 deficiencies was partially addressed by Lopez et al. [113], using human skin fibroblasts with a primary CoQ10 deficiency resulting from mutations in PDSS2, COQ2, and COQ9. Whilst not affecting cellular bioenergetics, the administration of vitamin C normalised oxidative-stress-induced cell death, suggesting complementary supplementation with antioxidants such as vitamins C or E may be of benefit in patients. To date, there have been no clinical studies supplementing antioxidants (other than CoQ10) such as vitamins C and E or alpha-lipoic acid in primary CoQ10 deficient patients, and such studies are now warranted.

## 6. Conclusions

1. As a result of the rarity of primary CoQ10 deficiency, there have been no randomised controlled trials to investigate the supplementation of CoQ10 in these disorders. However, from the data presented in the present article, we conclude that there is sufficient documentary evidence to support the use of supplementary CoQ10 for the treatment of primary CoQ10 deficiency, particularly for patients with nephrotic syndrome, in agreement with the conclusions of Alcazar-Fabra et al. [6].
2. It is vital that CoQ10 supplementation is initiated at the earliest possible time, before irreversible tissue damage has occurred. It is also important that any supplement used must be manufactured to pharmaceutical standards and be of documented bioavailability in human subjects.
3. There are many questions relating to the metabolism of CoQ10 which are of relevance to the treatment of primary CoQ10 deficiency. These include whether the bioavailability of supplemental CoQ10 could be improved following administration via alternative routes; whether CoQ10 can cross the blood–brain barrier or placental barrier in man, and whether the co-administration of additional antioxidants might improve patient outcomes. However, a possible caveat to consider is that the particular gene affected or the location of the mutation may also have a bearing on the patient’s response to CoQ10 supplementation.
